# Bond Strength and Failure Pattern of Orthodontic Tubes Adhered to a Zirconia Surface Submitted to Different Modes of Application of a Ceramic Primer

**DOI:** 10.3390/ma12233922

**Published:** 2019-11-27

**Authors:** Francisco da Silva Araújo Milagres, Dauro Douglas Oliveira, Giordani Santos Silveira, Emanuelle de Fátima Ferreira Oliveira, Alberto Nogueira da Gama Antunes

**Affiliations:** 1 Graduate Program in Orthodontics, Department of Dentistry, Pontifical Catholic University of Minas Gerais, 30535-901 Belo Horizonte, Brazil; francisco.milagres@yahoo.com; 2 Postgraduate Program in Clinical Dentistry, Department of Dentistry, Pontifical Catholic University of Minas Gerais, 30535-901 Belo Horizonte, Brazil; giordanisilveira@hotmail.com (G.S.S.); emanuelleortodontia@gmail.com (E.d.F.F.O.); antunes1978@gmail.com (A.N.d.G.A.)

**Keywords:** ceramics, shear strength, dental bonding, dental acid etching

## Abstract

The aim of this study was to evaluate the shear bond strength of orthodontic tubes adhered to ceramics with the Transbond™ XT bonding resin (3M, Maplewood, MN, USA) while varying the surface treatment. Then, the adhesive remaining index (ARI) was verified, and the representative fracture patterns were evaluated via scanning electron microscopy. Forty-eight zirconia blocks were divided into three groups, varying the number of layers of the 10-methacryloyloxy-decyl dihydrogen phosphate (MDP) primer: one, two, or three applications. In addition, 16 lithium disilicate IPS E.max ceramic disks (Ivoclar Vivadent, Schaan, Liechtenstein) were conditioned with 10% hydrofluoric acid for 20 s and underwent a single-layer primer application regimen. The four groups were further stratified to undergo bond testing after either 24 h (control) or 5000 cycles in a thermocycling machine. A shear bond strength test was performed (0.5 mm/min), and the MPa values obtained were submitted to a two-way analysis of variance and Tukey’s test. There was no statistical difference among the control group ceramics that received the varying surface treatments. After thermocycling, it was verified that both the E.max disks and the zirconia ceramics with three primer applications obtained the highest bond strength values. In the 24 h groups, a total displacement of the resin from the orthodontic tubes was observed (ARI of 1). After thermocycling, the highest prevalence of an ARI of 5 (adhesive failure) was observed among the zirconia ceramics with single-coat primer application, followed by those with triple-coat primer application (mixed failure). Three applications of the MDP-containing ceramic primer achieved the best result in the present study. Zirconia surface should be treated with three coats of MDP primer to achieve a level of bond strength similar to silica-rich phase ceramic.

## 1. Introduction

Zirconia is widely used as a ceramic crown material in dentistry. It has superior aesthetic appearance in comparison with metallic restorations and high mechanical strength in relation to other ceramic materials [1,2]. Nowadays, this type of ceramic may be used as computer-aided design/computer-aided manufacturing block materials to produce indirect restorations [3]. When an adult patient requires orthodontic treatment, it may be necessary to bond orthodontic appliances on a zirconia surface, especially on zirconia crowns of posterior teeth.

Despite providing structural strength, this ceramic’s high crystalline phase makes it harmless to hydrofluoric acid [4,5,6,7]. Some studies have suggested surface treatments must be performed via the impregnation of silica [8] (tribochemical treatment), sandblasting with aluminum oxide particles [9], or creating surface roughness with diamond burs [9,10]. However, the creation of irregularities on the surface tends to lead to microcracks [11]. These cracks accumulate tensions and act as a starting point for their growth and propagation, decreasing the clinical performance of indirect restorations [10,11]. Furthermore, using a 3-trimethoxysilane-propyl methacrylate (silane) coupling agent has no effect over these ceramic surfaces, because there is no silicon-rich glassy phase for covalent bonding with silane [12].

Notably, the use of primers or resin cements containing 10-methacryloyloxy-decyl dihydrogen phosphate (MDP) may increase zirconia bond strength between zirconia ceramics and resin materials [13,14,15,16,17,18,19]. Furthermore, this approach represents a passive way of modifying the ceramic without blasting, abrasive burr application, or any other form of mechanical treatment. MDP is an acid phosphate-based monomer that has an affinity for metal oxides, creating conditions for the resin cement to bond on this type of surface [20,21]. Additionally, MDP primer has been used as a treatment on zirconia surfaces aiming to bond orthodontic devices [22].

Even with this idea consolidated in the literature, there is not yet any definitive protocol for the stable chemical treatment of zirconia, as in the case of ceramics containing silica, which may be treated with hydrofluoric acid and silane [23], or in cases of zirconia ceramics covered by silica-rich phase [24]. Thus, this study sought to evaluate the effects of the application mode of a primer specially developed for ceramic surfaces, rich in silica or not, on the bond strength of orthodontic tubes adhered to a zirconia-based ceramic. In addition, the effects of thermocycling were tested, and the failure mode was evaluated. The hypotheses tested were that the application of more than one primer coat would maintain the bond strength values after the samples were thermocycled and that there would be no differences in the fracture patterns between the tested groups.

## 2. Materials and Methods

The materials are described in Table 1. A total of 48 blocks (1.5 mm × 7.0 mm × 7.0 mm) of Zirkonzahn Prettau (Zirkonzahn GMBH, Gais, Italy) and 16 disks (7 mm in diameter and 1.5 mm thick) of disilicate ceramic (IPS E.max; Ivoclar Vivadent, Schaan, Liechtenstein), included with acrylic resin in 2-inch polyvinyl chloride joints (Amanco; Mexichem, São Paulo, Brazil), were used. Prior to bonding, all specimens were sandblasted (Soflex; 3M, Maplewood, MN, USA) to remove any remaining acrylic resin on the surfaces. Next, they were polished with pumice slurry and washed and dried with an air jet until no wet surfaces were seen. Then, 64 stainless steel orthodontic tubes (Infinity Tube; Hangzhou Xingchen, Dental Instrument & Material Co. Ltd, Hangzhou, China) were used for bonding.

The 16 IPS E.max specimens, previously polished with a Sof-Lex™ disk (3M, Maplewood, MN, USA), pumice stone, and water, were conditioned with 10% hydrofluoric acid for 20 s and washed thoroughly with air–water jets. A layer of ceramic primer air-drying was applied, and tubes were subsequently bonded with Transbond™ XT resin (3M, Maplewood, MN, USA). Any excessive resinous material was removed with the aid of an exploratory probe, and the assembly was photopolymerized (Valo; Ultradent, South Jordan, UT, USA) with a light intensity of 1000 mW/cm^2^, with application lasting 10 s on each side (mesial and distal). This ceramic was used, because it has SiO_2_ in its composition, which is affected by the presence of 3-MPS in the composition of the ceramic primer.

Separately, the 48 zirconia specimens were randomly divided into three groups of 16 each. In the first group, the second molar tubes were bonded as recommended by the primer manufacturer (Clearfill ceramic primer; Kuraray, Tokyo, Japan). The zirconia received a layer of the primer and was dried with an air jet. The tubes were then bonded using the same Transbond™ XT resin, the excess was removed with the aid of an exploratory probe, and the specimens were photopolymerized in the same manner as previously described. In the second group of 16 zirconia specimens, the second molar tubes were bonded after the same preparation process outlined in the first group, but two layers of primer were applied before bonding and dried with air jets between the applications and immediately prior to bonding. Thereafter, the tube was bonded in the same manner as described above. Finally, in the third group of zirconia specimens, the tubes were bonded in the same way as in the previous two zirconia groups but with three layers of primer applied and dried with air jets between the applications and immediately before bonding. Again, the tubes were bonded in the same manner as described above.

Next, each of the four 16-specimen groups were randomly divided into two groups of eight specimens each. The groups underwent either a bond strength test 24 h after the bonding (control) or after 5000 cycles in a thermocycling machine (5 °C and 55 °C for 30 s each) following the bonding, as presented in Table 2.

The specimens were then evaluated using a shear test device (Odeme; Luzema, Santa Catarina, Brazil). The device was attached to the bottom of a universal testing machine (Emic Model 500; São José dos Pinhais, Paraná, Brazil). A chisel-shaped beveled stainless-steel tip was attached to the 5 kilonewtons (kN) load to test the shear bond strength between the ceramic and the base of the orthodontic tube. A crosshead speed of 0.5 mm/min was used to test the shear bond strength, thereby developing a degree of shear stress at the tube–resin base interface until the moment of detachment failure. The values were recorded directly in N on the digital monitor of the testing machine. Subsequently, the value in N of each specimen was divided by the internal area of the bracket (13.5 mm^2^), leading the results to all be expressed as sheer bond strength in megapascal (MPa) values. The groups described in Table 2 were tested 24 h after bonding and after artificial aging with 5000 cycles (5 °C and 55 °C). The MPa results were submitted to a two-way ANOVA and Tukey’s test under a 95% confidence level.

After failure, all of the specimens were examined under a 10× magnification optical microscope (SZ61; Olympus, Tokyo, Japan), and the amount of resin remaining was classified according to a “Resin Index” (Table 3) [14]. A representative specimen of each type of fracture found in the study was examined using scanning electron microscopy (JSM-IT 300; JEOL Ltd., Tokyo, Japan). For this, each selected ceramic surface was stored for 2 h inside a silica gel-containing vessel and then gold-sputtered (Denton Vacuum, DESK V-STANDARD model; JEOL Ltd., Tokyo, Japan) for 3 min under 20 mAmps, creating a film thickness of 195 angstrom (Å). These samples were observed with a voltage acceleration of 20 kv, work distance of 14 mm, and a spot size 55 nm, under high vacuum.

## 3. Results

The two-way ANOVA revealed differences both among the modes of surface treatment and between the 24 h period (controls) and those who had thermocycling aging (*p* < 0.05). The Tukey’s test results (shown in Table 4) indicated where the differences were. In the 24 h period, all groups showed no statistical differences. Then, after the thermocycling, it was observed that the E.max and zirconia triple-coat groups presented the highest values of shear bond strength. The lowest value was found for the zirconia single-coat group, being statistically different from the other groups.

**Figure 1:**
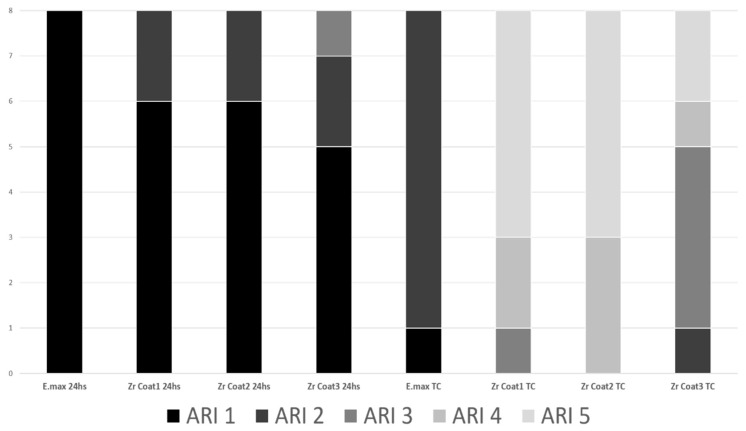
ARI of the fractured orthodontic tubes.

Figure 1 presents the failure pattern according to the ARI criteria (Table 3). The E.max control subgroup had all samples fractured at an ARI classification of 1, characterized as when the resin cement detached from the orthodontic tube inner surface. After thermocycling, the E.max subgroup was the only one to show some ARI 1; most of the rest of the specimens were ARI 2. Only zirconia treated with the ceramic primer and subjected to artificial aging by the thermocycling method showed ARI 5 and 4 classification.

**Figure 2:**
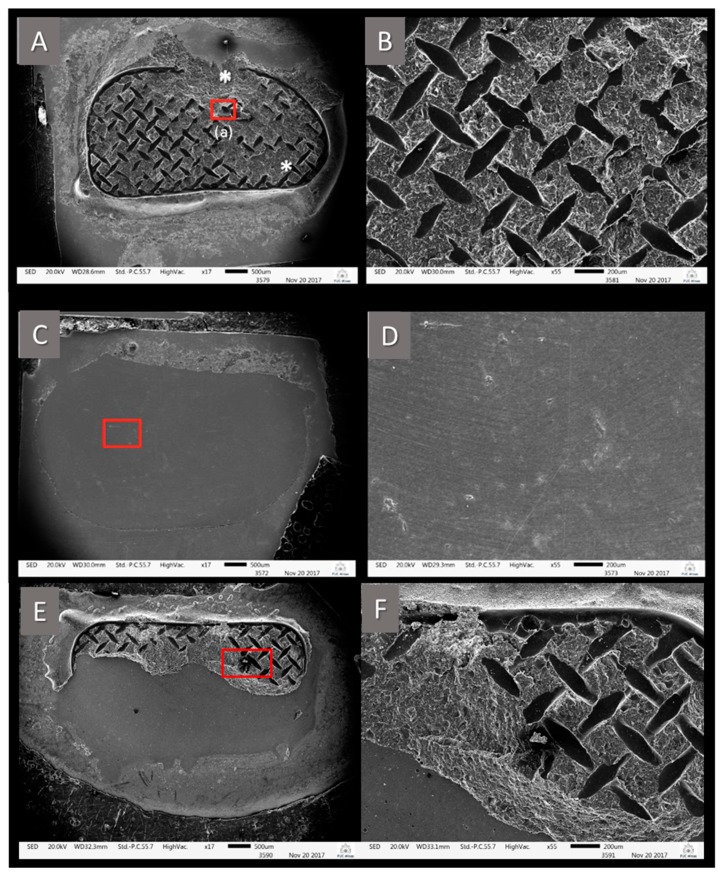
SEM images showing different failure patterns. (**A**) (17× magnification) shows almost the entire interior design of the orthodontic tube used. The structure of the resinous material remained almost entirely on the ceramic, except for the region marked with *. (**B**) (55× of magnification) represents the region marked by the red square at (**A**). This fracture is classified as ARI 1, 100% of the resin attached to the zirconia. (**C**) (17× of magnification) is representative of the ARI 5 (no resin over the zirconia). In its high magnification (**D**) (55× of magnification) of the region marked with the red square, zirconia is free of resinous material. (**E**) (17× of magnification) represents the fracture pattern ARI 3, 10%–89% of the resin material attached to the ceramic. The image shows a mixed failure mode, as there was a displacement of the resinous material from the orthodontic tube, cohesive fracture of this same resinous material which is visualized in image (**F**), (55× magnification), and there is ceramic free of any resinous remnant.

Figure 2 shows the most frequent failure indices observed during optical microscopy. Figure 2A,B displays an image of a zirconia ceramic surface, representative of the ARI value of 1 classification in SEM. In Figure 2A, all cement remained on the ceramic after the fracture test, while the orthodontic tube was completely detached from the cement. The asterisk (*) in Figure 2A indicates a region where there is a discontinuity of the orthodontic tube mesh impression, which is more visible in Figure 2B due to a magnification of 55×. This indicates some cohesive failure of the resin cement or that impregnation of the metallic mesh did not occur. Figure 2C shows an SEM image of a zirconia ceramic surface representative of the ARI value of 5 classification, in which there was complete adhesive failure of the Transbond™ XT resin. In Figure 2D, it is possible to visualize in more detail the surface of the zirconia ceramic, free from any residual adhered cement. Differently, Figure 2E indicates the ceramic surface of a zirconia ARI 3 classification example. It can be noted that part of the Transbond™ XT resin remained on the cement. Figure 2F reveals a mixed appearance of the failure, with some cohesive fracture and adhesive detachment from the inner surface of the orthodontic tube.

## 4. Discussion

This study verified that the use of the ceramic primer on zirconia surface attained bond strength values comparable to the control group, although the thermocycling procedure decreased their shear bond strength value. Thus, the hypothesis we proposed can be partially accepted, because, within the group that underwent 5000 cycles of water at 5 °C and 55 °C, the zirconia specimens with three layers of primer achieved values equal to those of the E.max ceramic specimens treated with hydrofluoric acid and the ceramic primer product. This study used different numbers of applications of a single primer in order to elucidate if a simpler surface treatment method can be used without the risk of creating microscopic defects or without impairing the aesthetics of the vestibular surface of the restorations.

Previously, Kern and Thompson (1995) compared the effects of different surface treatments on ceramics [12]. The idea behind the use of metal primers was justified by their main molecule feature, a bifunctional monomer that acts on the metallic oxide layer of non-precious metal alloys and which has the ability to copolymerize with the materials [3,13,20]. Nevertheless, in 2004, the use of blasting was questioned, because this treatment can create cracks in the surface of ceramics reinforced with metallic oxides [10]. Therefore, the ceramics of the present study were not subjected to any type of blasting.

The ceramic primer used was a blend of ethanol solution (nearly 80% by weight), MDP, and a quantity of less than 5% by weight of silane. This material was initially designed to treat different types of ceramics, including those rich in silica, alumina-reinforced, or composed almost entirely of zirconia [20,24,25]. The ceramic primer product promoted an increase in bond strength values, although not to a point where it prevented the thermocycling effects [1]. These findings agree with the results of the present study. The minimum value of shear strength is in the range of 5.9–7.9 MPa [26]. Thus, all groups obtained adequate bond strength values able to sustain a static load, wet environment, and temperature variation. The artificial aging caused by thermal variation with 5000 cycles, however, reduced the values of all of these parameters.

After thermocycling, the E.max ceramic had its strength value lowered by only 4.3%, while the zirconia triple-coat group showed a reduction in bond strength of 18.5%, the zirconia double-coat group experienced a reduction of 33.8%, and the zirconia single-coat group presented a reduction of 71%. Of note, the thermocycled E.max and zirconia double-and triple-coat specimens had bond strengths that remained sufficiently efficient enough to comply with the value established by Reynolds in 1975 [27], although there was a statistically significant difference between the E.max and zirconia double-coat groups. These results indicate that a ceramic with a high amount of zirconia should be treated with three ceramic primer applications to exhibit a level of performance comparable to that of a ceramic containing silica in its composition. In fact, Prettau zirconia boasts a small amount of silica (Table 1), and that may contribute to good bond strength values when more coats of ceramic primer were applied on the zirconia. Similarly, the idea is that there was more MDP available to act on the zirconia. The same ceramic primer used in the present work was used to treat zirconia blocks in a previous study [28]. In that study, the authors applied a thin layer of the ceramic primer on a sandblasted zirconia, dried with gentle air stream prior the Transbond™ XT adhesive primer. After thermocycling of 10,000 cycles, there was no statistical difference between this group and its control, with no thermocycling. Thus, because the orthodontic treatment is continued for a long period, it is preferable to apply the 10-MDP-containing ceramic primer to the sandblasted zirconia, followed by application of the orthodontic primer [28].

The MDP is capable of establishing a very intensive and stable chemical interaction with hydroxyapatite [29]. Additionally, the MDP molecule has been shown to chemically bond with the outermost surface of zirconia [14,15,16,17,18,19]. The MDP molecule tends to remain with its adhesive group, the one containing the zirconia-oriented phosphate moiety, while its polymerizable group is oriented toward other resin monomers [15]. The molecule will remain in contact with zirconia through hydrogen bonding or ionic interaction, P-OH and Zr-OH, or between P-O and partially positive Zr [8]. Even MDP that is present in universal adhesive bottles has an effect on improving the adhesion of resinous materials to zirconia ceramics when shear bond strength was evaluated [16,24]. The chemical treatment, either primer or adhesive application, affects the surface topography and its surface chemistry properties (roughness, wettability, and surface free energy of zirconia) [19]. The resins often used in adhesion procedures are challenged when long-term water storage artificially age the material by creating conditions for a slow water sorption and degradation of the structure of the resin materials [30,31]. This phenomenon could increase the chance of loss of the union of the orthodontic device, bracket, or orthodontic tube.

It should be noted that, at each primer application in the present study, a solvent evaporation procedure with a continuous air-jet application of 10 s was performed. This airflow allowed for the evaporation of any residual ethanol, which, according to the manufacturer, is present in up to 80% by weight in the solution. In dentin, the excess solvent leads to the formation of a hybrid layer, and the adhesive layer becomes more hydrophilic and susceptible to water absorption over time. This leads to the softening of the polymers and, consequently, the breaking of the long polymer in smaller chains, which make the resin materials more prone to crack propagation [32].

Thermocycling in water baths has been used to simulate the aging effect on resin materials [21,30]. The artificial aging induced by thermocycling accelerates the hydrolysis of interface resin components and leads to contraction and expansion stresses [32]. In our study, the effect was enough to reduce the shear bond strength across all groups, even the one with three applications of Ceramic Primer over the zirconia. Clearly, the values of the reduction seem different for each group. The zirconia with single-coat application showed the greatest change (i.e., reduction) between the 24 h control subgroup and the thermocycling subgroup, while the zirconia with triple-coat application experienced the least reduction between its two subgroups among the groups treated with MDP primer (Table 4). A possible explanation is that the zirconia with single-coat application specimens experienced more water uptake during the thermocycling, because this group had a greater number of adhesive failures (Figure 2). This suggests that the water entered more freely between the Transbond™ XT resin and the ceramic surface, thus breaking the union between these two surfaces much more easily. This may somehow explain the lowest bond strength value of 5.05 MPa and the highest number of adhesive failures recorded in this group (Figure 2).

Further studies are needed to evaluate different surface treatment methods. A limitation of the present study was to use only one zirconia ceramic and only one type of ceramic primer. Future studies may take advantage of other varieties of products or even treat ceramics with different universal adhesives. Additionally, different aging methods can be used to challenge the bond between ceramics and resinous materials, such as long-term storage in water at 37 °C, thermocycling greater than 5000 cycles, and use of mechanical cycling.

## 5. Conclusions

Within the limitations of this study, it is possible to conclude that thermocycling decreased the shear bond strength of all test groups, including one containing a silica-rich ceramic. It is recommended that ceramics composed essentially of zirconia must be treated with at least three coats of MDP primer so as to exhibit a bond strength comparable to that of a silica-rich ceramic treated with hydrofluoric acid and silane. This implies a more conservative procedure and an alternative to mechanical treatments that may harm the labial surface of the ceramics.

## Figures and Tables

**Table 1:** Materials used in this study.

Material	Type	Composition	Manufacture
**Clearfil Ceramic Primer**	Ceramic primer	3-MPS, ethanol, 10-MDP	Kuraray, Tokyo, Japan
**Transbond™ XT**	Composite resin	Silane-treated quartz, silane treated silica, Bis-GMA, bisphenol-a-bis (2-hydroxyethylether) dimethacrylate, diphenyliodonium hexafluorophosphate	3M Unitek; Monrovia, CA, USA
**Zirconia Prettau**	Ceramic	ZnO_2_, SIO_2_, Al_2_O_3_, Y_2_O_3_	Zirkonzahn Prettau, Gais, Italy
**IPS E.max Press**	Lithium disilicate Ceramic	SIO_2_, LI_2_O, K_2_O, MgO, ZnO_2_, Al_2_O_3_, P_2_O	Ivoclar Vivadent, Schaan, Liechtenstein

**Table 2:** Design of the groups (n = 8).

Control	Thermocycling
E.max 24 h	E.max TC
Zr coat 1 24 h	Zr coat 1 TC
Zr coat 2 24 h	Zr coat 2 TC
Zr coat 3 24 h	Zr coat 3 TC

**Table 3:** Adhesive resin remaining index (ARI).

ARI	Criteria
1	100% of the resin was attached to zirconia
2	90% of the resin was bonded to zirconia
3	10%–89% of the resin was attached on the zirconia
4	Less than 10% of the resin was attached on the zirconia
5	No resin was present on zirconia surface

Adapted from Artun; Bergland (1984) [25].

**Table 4:** Shear bond strength values means (MPa) and standard deviation in parentheses.

Ceramic	24 h	Thermocycling
E.max	19.13 (1.06) Aa	18.82 (2.30) Aa
Zr coat 1	17.36 (3.43) Aa	5.05 (1.70) Cb
Zr coat 2	17.13 (2.25) Aa	11.33 (5.43) Bb
Zr coat 3	18.42 (0.74) Aa	15.00 (1.22) ABb

MPa values followed by different capital letter indicate statistical difference in columns by Tuckey test (*p* < 0.05). Similarly, values followed by different lower-case letters indicate statistical difference in rows.
